# Supplementary material for: Human and mouse neutrophils share core transcriptional programs in both homeostatic and inflamed contexts
Source: Nat Commun. 2023 Dec 8;14:8133. doi: 10.1038/s41467-023-43573-9 (PMC10709367; doi:10.1038/s41467-023-43573-9)
Supplement: Supplementary file 1 — Supplementary Information [file 41467_2023_43573_MOESM1_ESM.pdf]

Supplementary Information

Human and mouse neutrophils share core transcriptional programs in both homeostatic and inflamed contexts

Nicolaj S. Hackert[1,2,4,7,*], Felix A. Radtke[1,2,5,8,9,*], Tarik Exner[1,2,*], Hanns-Martin Lorenz[1], Carsten Müller-Tidow[3,6], Peter A. Nigrovic[4,5], Guido Wabnitz[2], Ricardo Grieshaber-Bouyer[1,2,6,10,11]

1) Division of Rheumatology, Department of Medicine V, Heidelberg University Hospital, Heidelberg, Germany

2) Institute for Immunology, Heidelberg University Hospital, Germany

3) Department of Medicine V, Hematology, Oncology and Rheumatology, Heidelberg University Hospital, Heidelberg, Germany

4) Division of Immunology, Boston Children's Hospital, Harvard Medical School, Boston, MA, USA

5) Division of Rheumatology, Inflammation, and Immunity, Brigham and Women's Hospital, Harvard Medical School, Boston, MA, USA

6) Molecular Medicine Partnership Unit, European Molecular Biology Laboratory (EMBL), University of Heidelberg, Heidelberg, Germany

7) Broad Institute of MIT and Harvard, Cambridge, MA, USA

8) MRC Molecular Haematology Unit, MRC Weatherall Institute of Molecular Medicine, University of Oxford, Oxford, UK

9) Oxford Centre for Haematology, NIHR Oxford Biomedical Research Centre, Oxford, UK

10) Deutsches Zentrum für Immuntherapie (DZI), Friedrich Alexander Universität Erlangen-Nürnberg and Universitätsklinikum Erlangen, Erlangen, Germany

11) Department of Internal Medicine 3 – Rheumatology and Immunology, Friedrich Alexander Universität Erlangen-Nürnberg and Universitätsklinikum Erlangen, Erlangen, Germany

* Nicolaj S. Hackert, Felix A. Radtke, and Tarik Exner contributed equally to this work.

Correspondence:
Professor Dr. med. Ricardo Grieshaber-Bouyer
Department of Internal Medicine 3 – Rheumatology and Immunology
Friedrich-Alexander-Universität Erlangen-Nürnberg and Universitätsklinikum Erlangen
Glückstraße 4A
91054 Erlangen, Germany
ricardo.grieshaber@fau.de
www.rgb-lab.de

Supplementary Table 1. Human neutrophil flow cytometry panel.

List of antibodies/markers used to perform flow cytometry on human neutrophils.

Marker	Channel	Clone	Vendor	Catalog #	Dilution	Panel 1	Panel 2
**LIVE/DEAD**	Pacific Orange	N/A	BioLegend	423103	1:300	+	
**LIVE/DEAD**	BUV496	N/A	BioLegend	423107	1:300		+
**DAPI**	BUV496	N/A	Sigma-Aldrich	D9542	1:1000		+
**CD15**	APC-Cy7	W6D3	BioLegend	323047	1:100	+	+
**CD69**	BV421	FN50	BioLegend	310929	1:50	+	
**CD40**	Alexa Fluor 700	5C3	BioLegend	334327	1:50	+	+
**CD14**	PE-Cy7	M5E2	BD Biosciences	557742	1:100	+	+
**IL4R**	PE	G077F6	BioLegend	355003	1:100	+	+
**PD-L1**	APC	29E2A3	BioLegend	329708	1:20	+	
**CD101**	APC	BB27	BioLegend	331007	1:100		+
**CXCR4**	BV421	12G5	BioLegend	306517	1:100		+
**CD62L**	BV605	DREG-56	BioLegend	304833	1:100		+

Supplementary Table 2. Mouse neutrophil flow cytometry panel.

List of antibodies/markers used to perform flow cytometry on mouse neutrophils.

Marker	Channel	Clone	Vendor	Catalog #	Dilution	Panel 1	Panel 2
**LIVE/DEAD**	Pacific Orange	N/A	BioLegend	423103	1:300	+	
**LIVE/DEAD**	BUV496	N/A	BioLegend	423107	1:300		+
**DAPI**	BUV496	N/A	Sigma-Aldrich	D9542	1:1000		+
**Ly6G**	APC-Cy7	1A8	BioLegend	127623	1:100	+	+
**CD69**	BV421	H1.2F3	BioLegend	104527	1:100	+	
**CD40**	PE-Cy5	3/23	BioLegend	124617	1:100	+	+
**CD14**	PE-Cy7	Sa14-2	BioLegend	123315	1:100	+	+
**IL4R**	PE	I015F8	BioLegend	144803	1:100	+	+
**PD-L1**	APC	10F.9G2	BioLegend	124311	1:100	+	
**CD101**	APC	Moushi101	ThermoFisher Scientific	17-1011-82	1:100		+
**CXCR4**	BV421	L276F12	BioLegend	146511	1:100		+
**CD62L**	BV605	MEL-14	BioLegend	104437	1:100		+

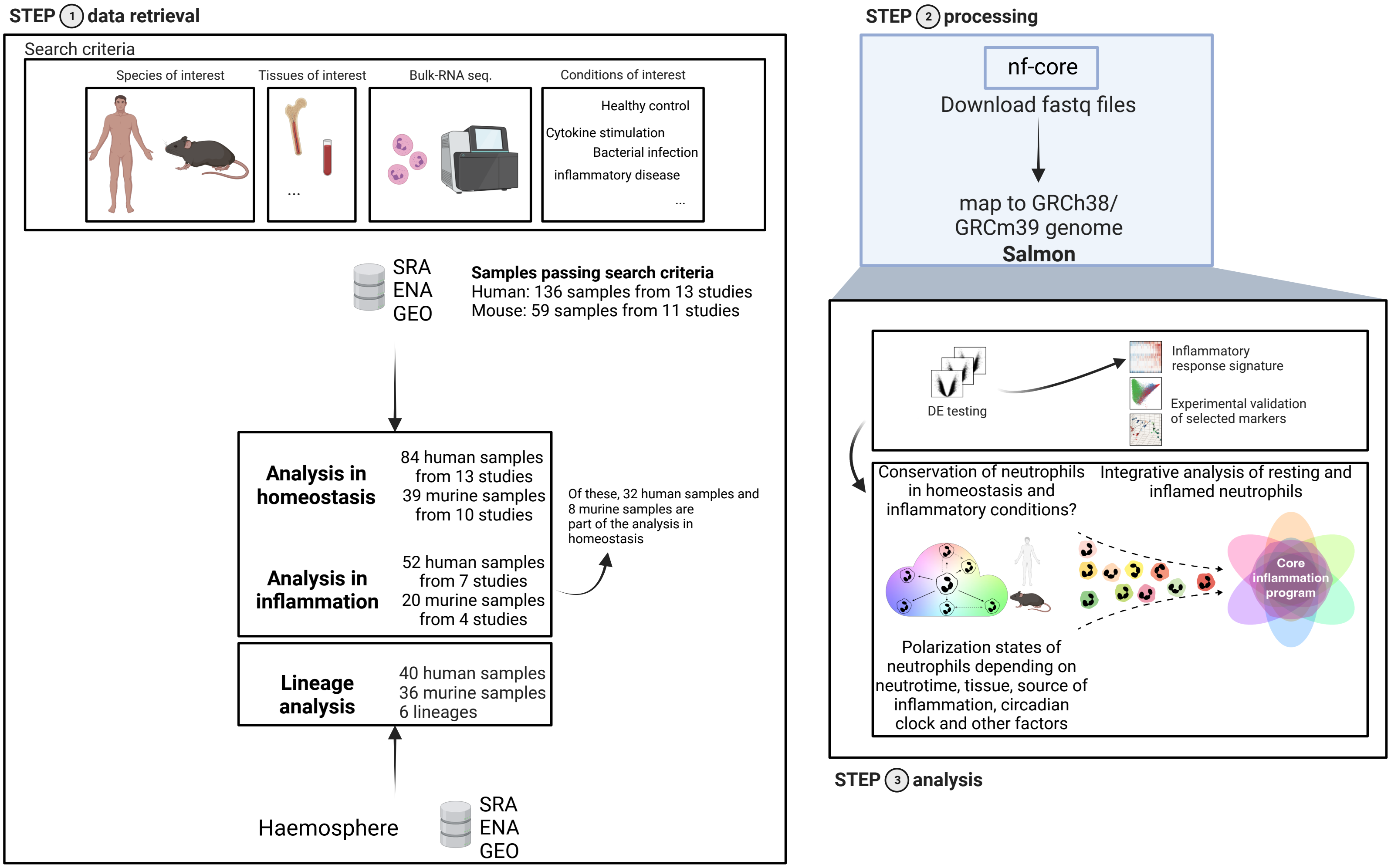

Supplementary Figure 1. Overview of the study.
Depiction of the search criteria for sample selection, processing steps, and analysis approach used in the study

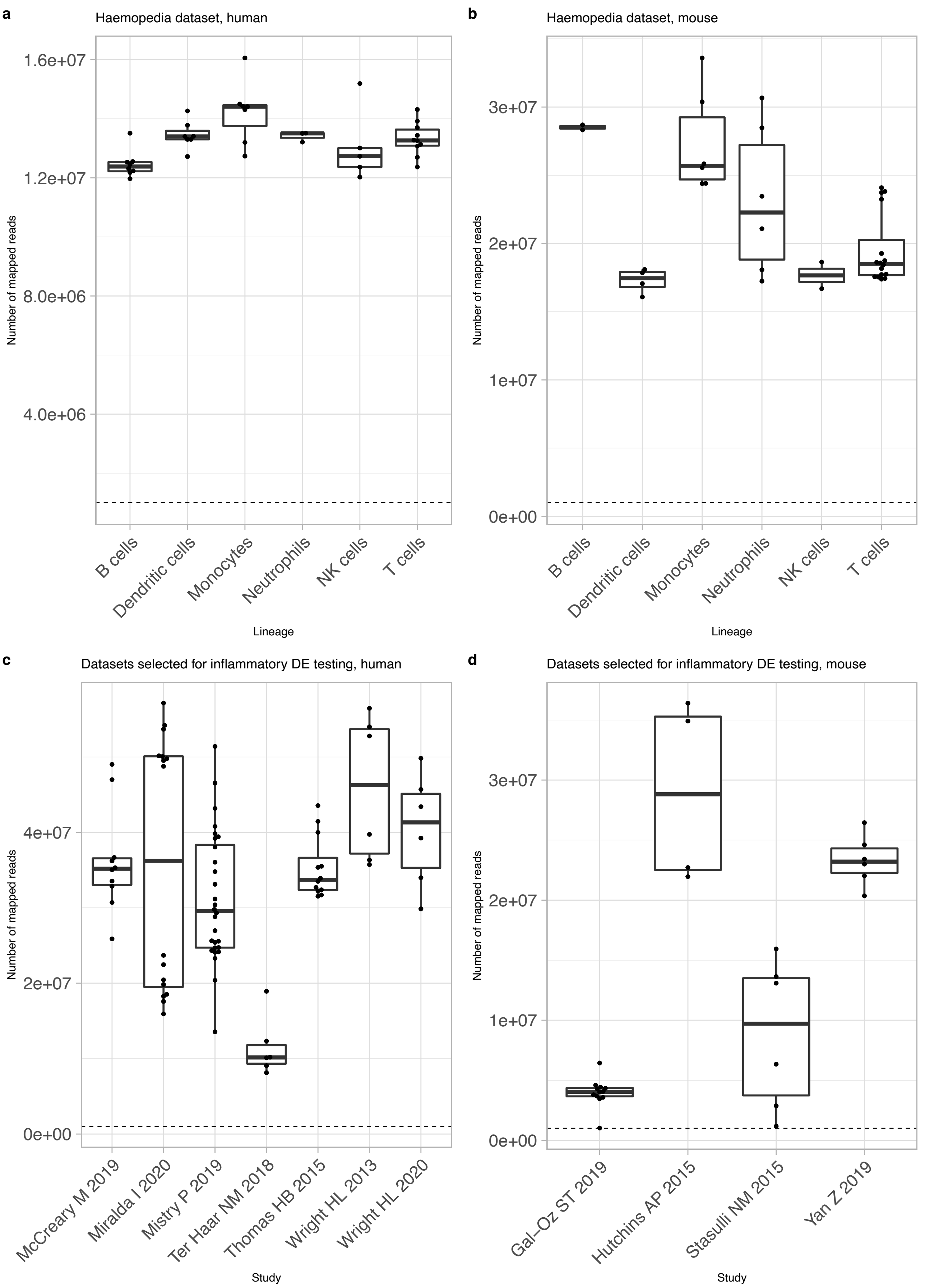

Supplementary Figure 2. Sequencing depths of individual libraries
Library size was defined as the number of mapped reads per sample and plotted. The dotted line represents 1 million mapped reads. **a** Library sizes of datasets from the Haemopedia atlas (human). **b** Library sizes of datasets from the Haemopedia atlas (mouse). **c** Library sizes of datasets used for differential expression testing (human). **d** Library sizes of datasets used for differential expression testing (mouse). Source data are provided as a Source Data file

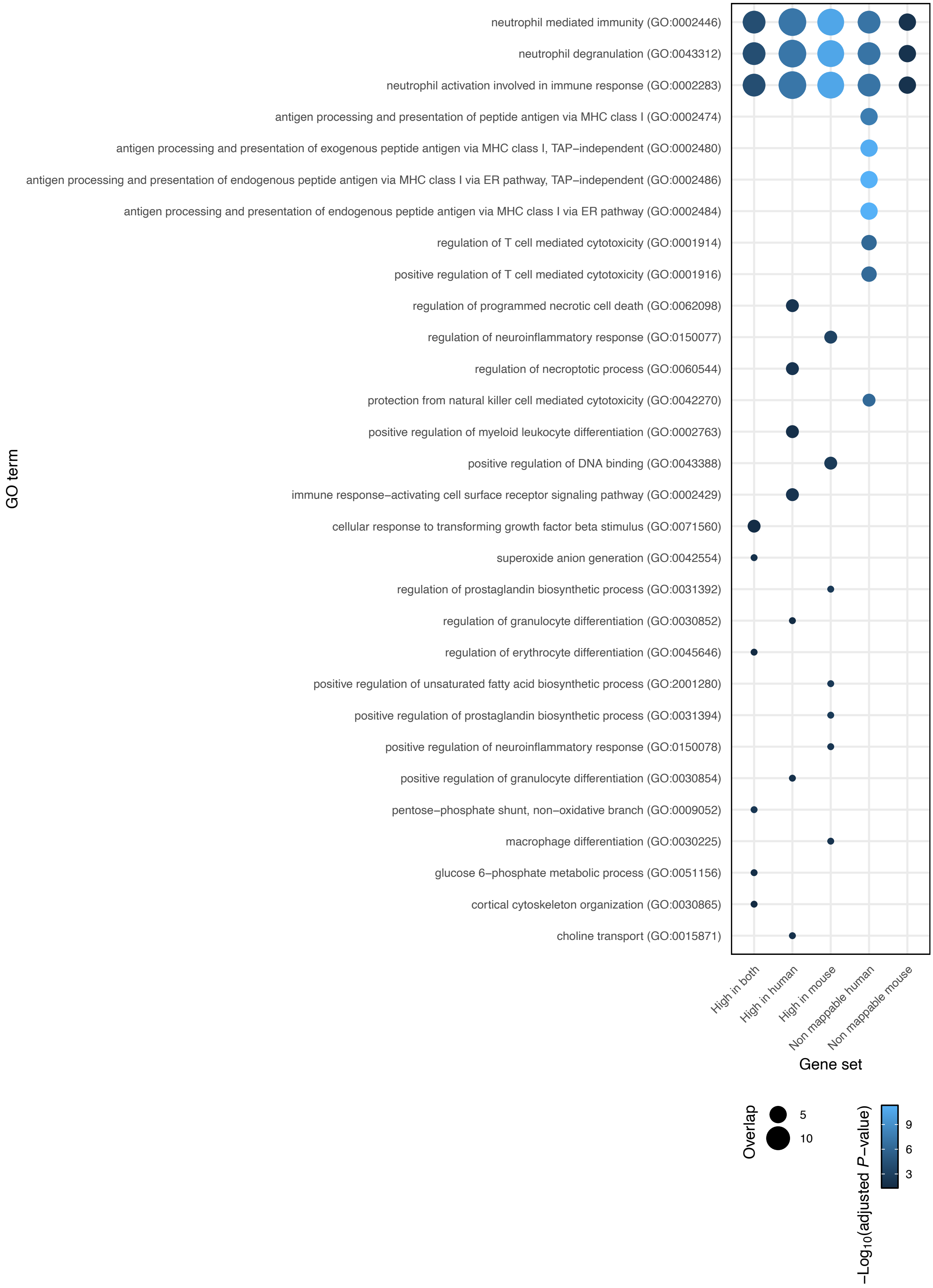

Supplementary Figure 3. Enrichment analysis for gene clusters in homeostasis.
Gene Ontology enrichment analysis results for five clusters showing different expression profiles between human and mouse neutrophils. Analysis was restricted to the "Biological Process" domain as defined by the Gene Ontology Consortium[1, 2]. Only significant enrichments are plotted as dots. Dot size corresponds to overlapping gene set size, color to the negative logarithm of adjusted *P*-values. GO terms are ranked according to their mean overlap across all significant hits from top to bottom. Source data are provided as a Source Data file.

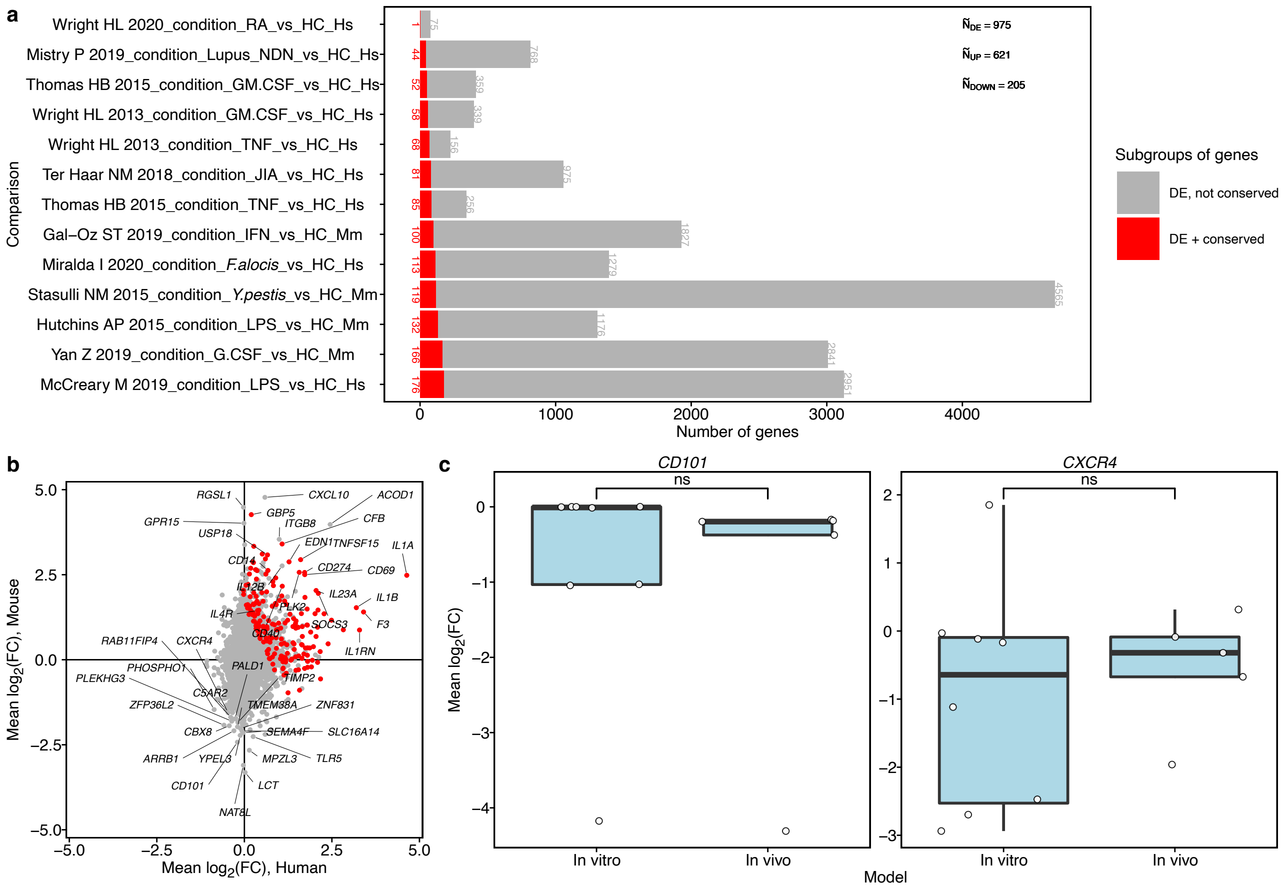

Supplementary Figure 4. Transcriptional diversity in inflamed neutrophils.

a Bar chart indicating the number of genes differentially expressed in each comparison (DE, not conserved), and the subset of genes that are differentially expressed and part of the core inflammation program (DE + conserved). Top right, median number of genes differentially expressed, and the subset of upregulated and downregulated genes. **b** Scatter plot of mean $\log_2$ fold changes in human versus in mouse from n=13 comparisons listed in **a**. Red, core inflammation program genes. We labeled the genes with the 20 highest and 20 lowest mean expression values as well as genes that were selected for validation (Fig. 6 and 7). **c** Individual $\log_2$ fold changes for *CD101* and *CXCR4* from n=13 comparisons listed in **a**. *P*-values have been retrieved from a Wilcoxon signed-rank test and adjusted using the Holm-Bonferroni method. Here, *in vitro* and *in vivo* models of inflammation were separated. Source data are provided as a Source Data file.

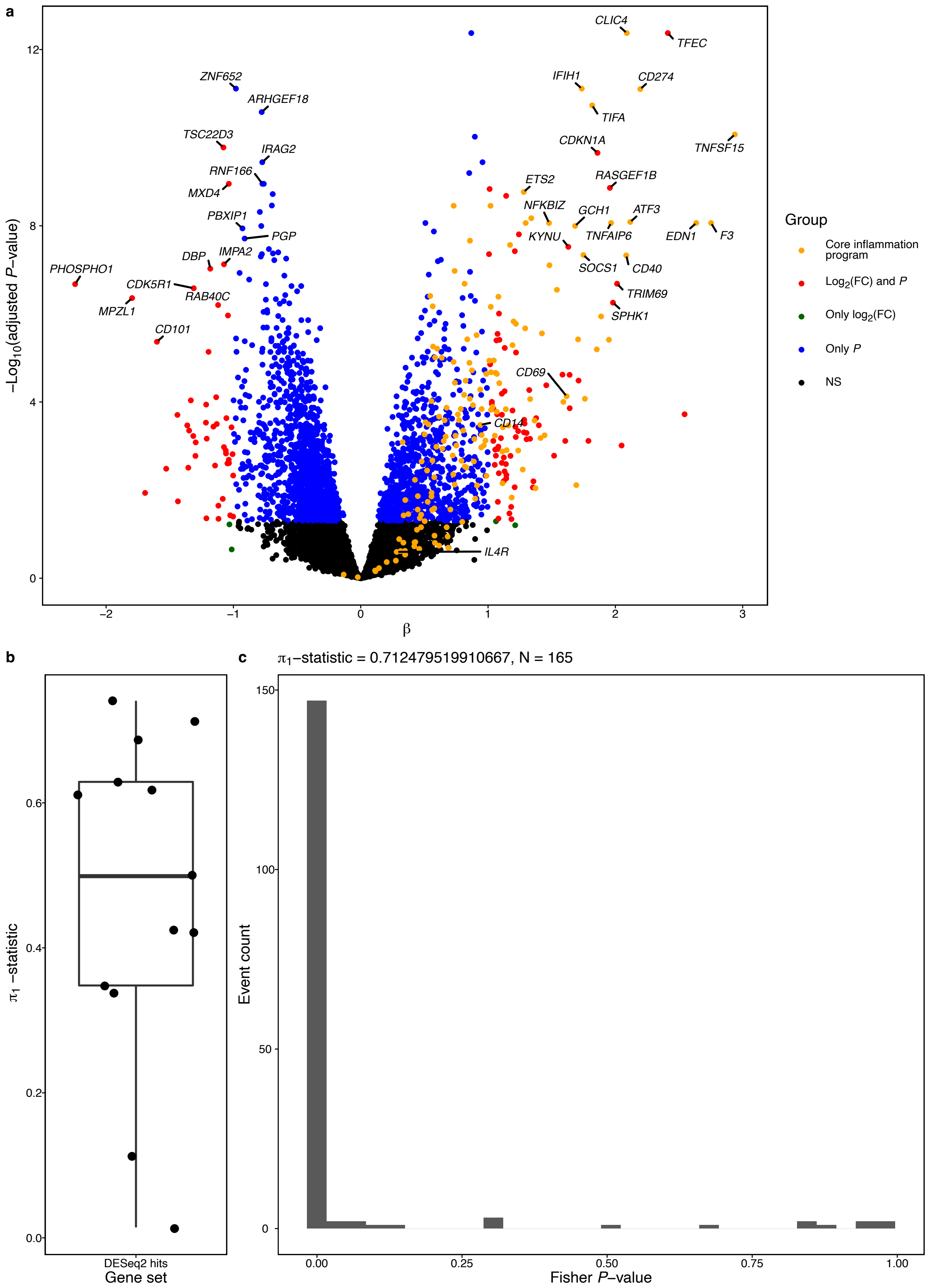

Supplementary Figure 5. High concordance of complementary linear mixed modeling approach with individual differential expression tests and fisher *P*-values.

a Volcano plot showing the differentially expressed genes according to a linear mixed model testing between healthy control and non-healthy samples. *N* = 141 upregulated, *N* = 42 downregulated genes in inflamed neutrophils. Core inflammation genes identified by Fisher's combined approach are highlighted in orange. *N* = 59 genes identified core inflammation genes pass the threshold in our linear mixed model and are shared. **b** Boxplot showing the π_1-statistic of top differentially expressed genes as identified according to the linear mixed model for each individual differential expression test. **c** Histogram showing an enrichment of low fisher *P*-values $\pi1 \approx 0.71$) for genes that are up- and downregulated according to the linear mixed model. Source data are provided as a Source Data file.

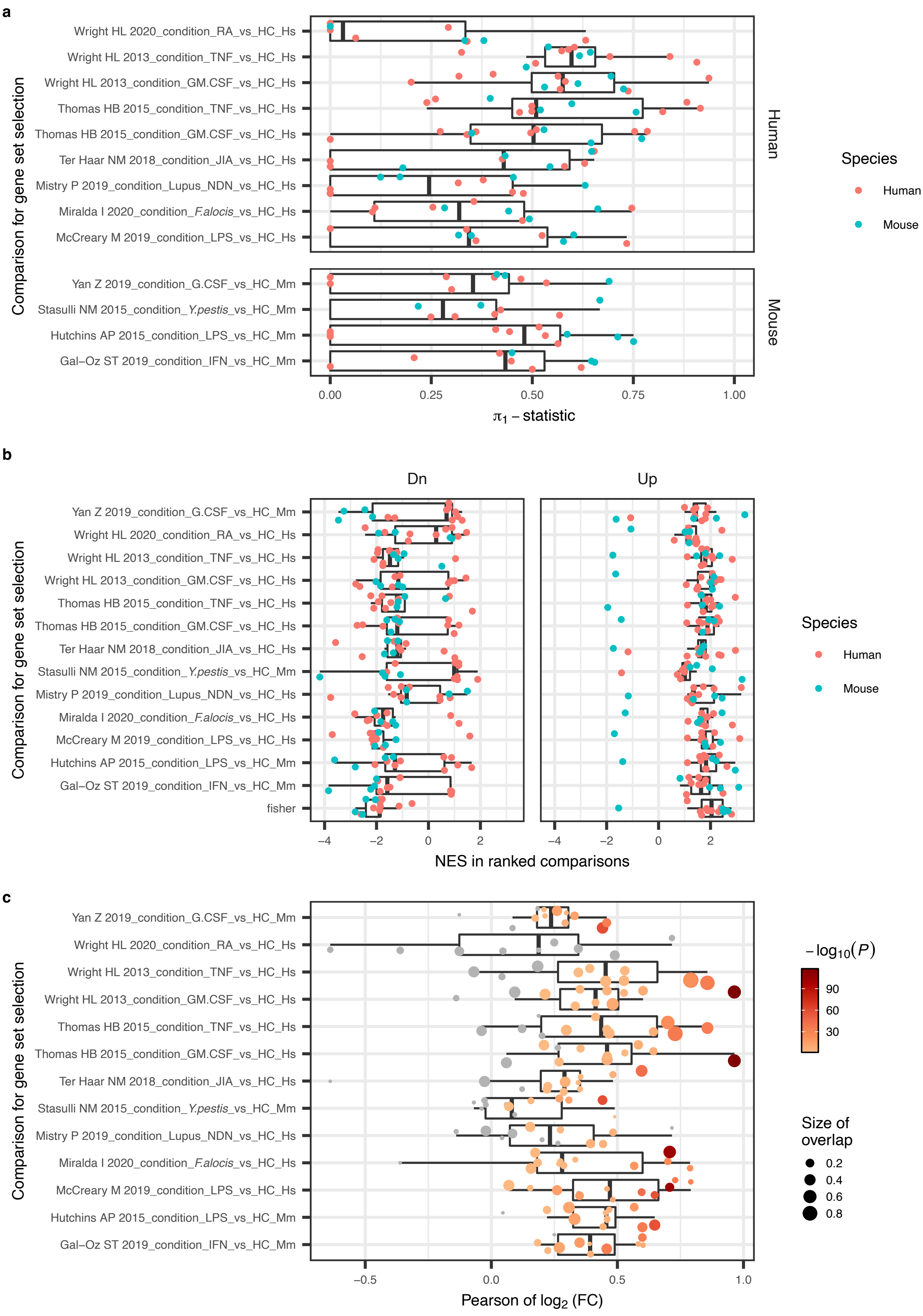

Supplementary Figure 6. Comparison of differential expression testing between individual studies.
a Boxplots depicting the π_1-statistic of all differentially expressed gene sets (Y-axis) in all other comparisons, respectively. Points are colored by species of the respective studies. **b** Enrichment scores of differentially expressed gene sets in each comparison (Y-axis) in the ranked differential expression lists of all other comparisons, respectively. Points are colored by species of the respective comparison. The entry "fisher" refers to the gene set derived from the Fisher's combined test as described in Fig. 3b and Methods. **c** Pearson's *r* of $\log_2$ fold changes of differentially expressed genes in each comparison that are also differentially expressed in other comparisons. Point size corresponds to overlap of differentially expressed genes, color to the negative logarithm of the *P*-value obtained from a Pearson product-moment correlation test. 6 correlations could not be determined due to a low overlap in differentially expressed genes. Source data are provided as a Source Data file.

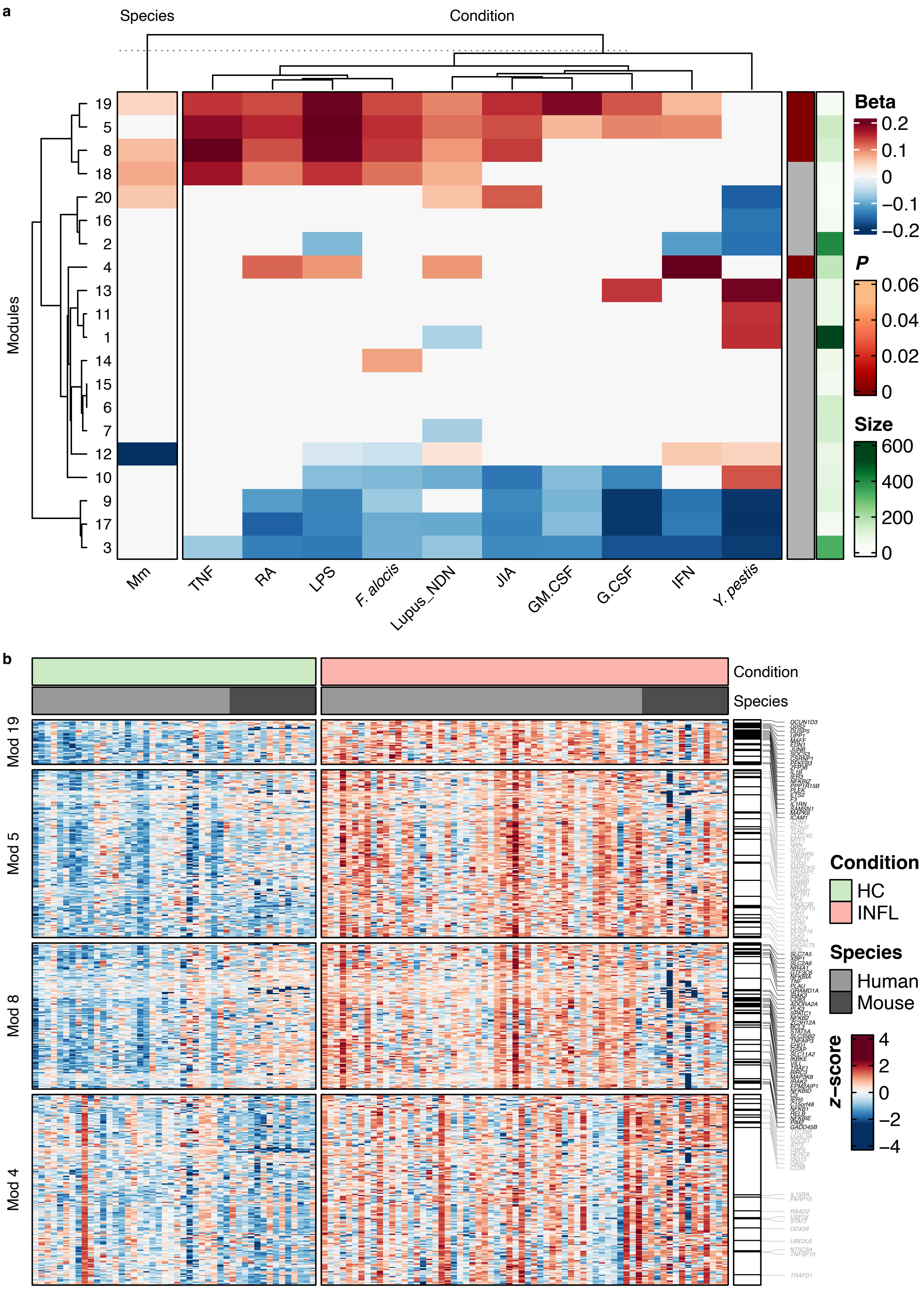

Supplementary Figure 7. WGCNA analysis across all studies used for differential expression analysis.
a Association of gene modules with each experimental condition and species. Eigengenes, a proxy of module expression, were modeled as a combination of condition and species for each module, using a linear model. The heatmap shows effect size estimates resulting from this modeling procedure. Effect size estimates resulting from non-significant associations were set to 0 to allow hierarchical clustering. For each module, a Fisher's exact test was performed to test for association with the core inflammatory response signature. Adjusted *P*-values are annotated on the right, gray indicates non-significance. Additionally, module size, defined as the number of genes assigned to that module, is annotated. **b** Relative expression profiles (*z*-score) of genes in modules that are significantly associated with the fisher core inflammatory response signature. Rows correspond to genes, columns to samples. Relative expression was calculated after batch correction for each study (ComBat). Black bars on the right of the expression profiles indicate gene membership in the fisher core signature. Source data are provided as a Source Data file.

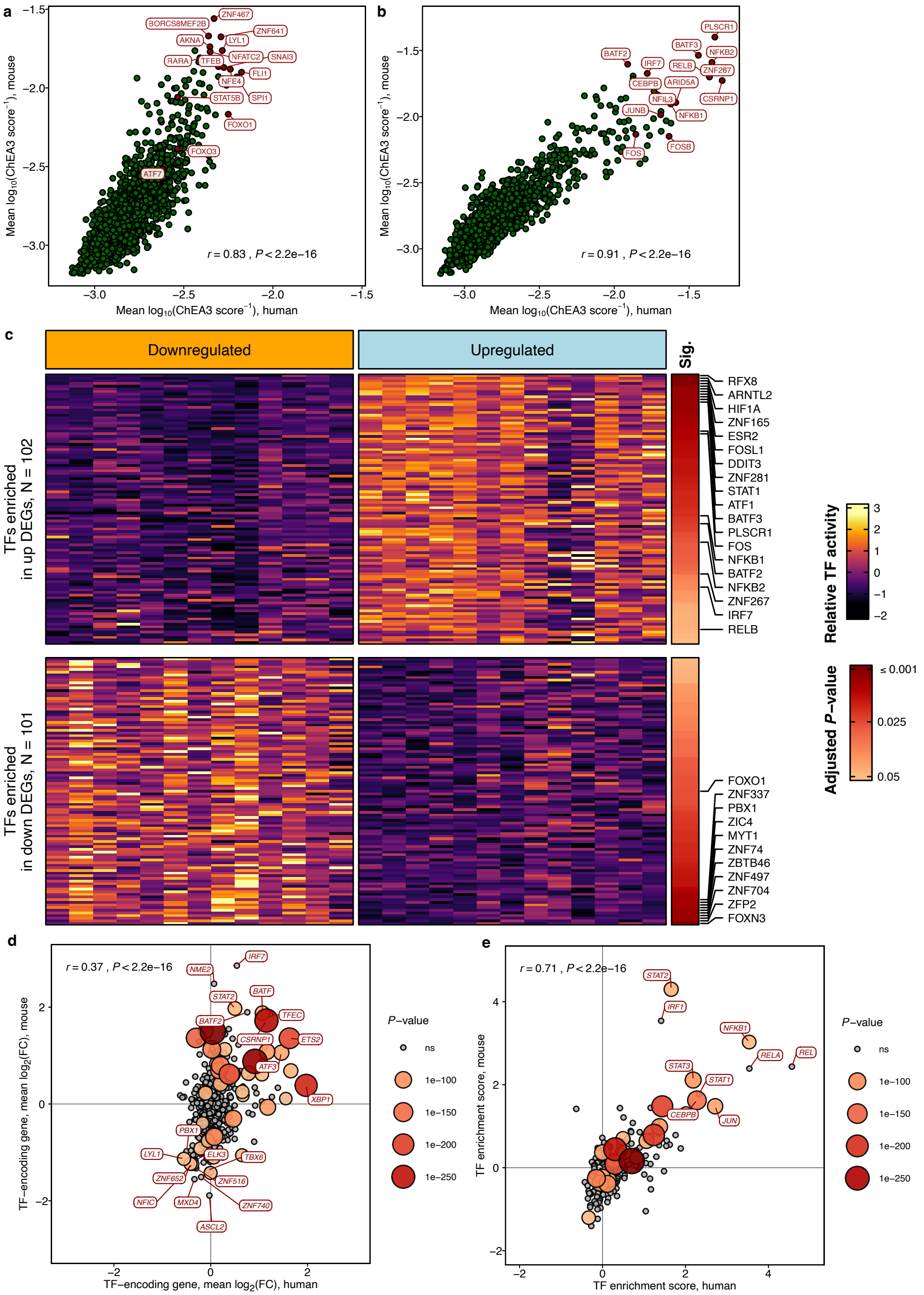

Supplementary Figure 8. Species-specific Transcription Factor Enrichment Analysis.
a Enrichment of transcription factors in genes associated with resting neutrophils is highly concordant between species. *N* = 250 genes with a negative $\log_2$(FC) in inflammation were sorted in ascending order of adjusted *P*-values for each comparison, and regulatory activity was derived as outlined in Methods. For each transcription factor, mean ChEA3-derived activity across human (x) and mouse (y) comparisons is shown; high values indicate high predicted activity. Top 10 transcription factors with the highest predicted activity as well as FOXO3, FOXO1, TFEB, RARA, STAT5B, and ATF7 are highlighted. **b** Enrichment of transcription factors in genes associated with inflamed neutrophils is highly concordant between species.
For each transcription factor, mean ChEA3-derived activity across human (x) and mouse (y) comparisons is shown; high values indicate high predicted activity. Top 10 transcription factors with the highest predicted activity as well as NFIL3, FOSB, FOS, CEBPB, and JUNB are highlighted. **c** Transcription factor enrichment in down- and upregulated genes in inflammation is shared across studies. Enrichment of transcription factors was calculated based on the top 250 down- (left) or upregulated (right) genes in each independent comparison in our differential expression analysis. The inferred activity of transcription factors (rows) is shown scaled across gene sets. Adjusted *P*-values from a *t*-test between inferred activity in down- vs. upregulated gene sets for each transcription factor are shown in the “Sig.” column. Transcription factors with the 10 lowest *P*-values per direction as well as those labeled in **a,b** were labeled. **d** Scatter plot of mean $\log_2$ fold changes per species for TF encoding genes. The collection of TF-encoding genes was retrieved from DoRothEA[3] (v1.8.0). Genes with the 10 highest and 10 lowest sums of expression were labeled. **e** Scatter plot of transcription factor enrichment scores calculated per species using decoupleR[4] (v2.2.2). The transcription factors with the 10 highest scores were labeled. Source data are provided as a Source Data file.

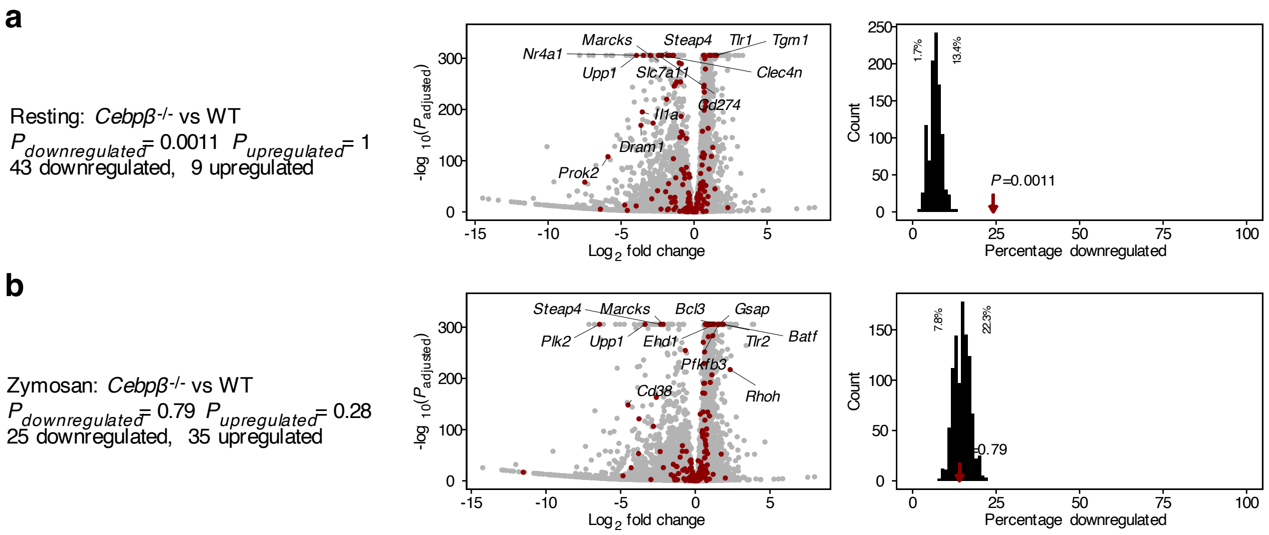

Supplementary Figure 9. Core inflammation genes show preferential downregulation after Cebpb knockout in mouse zymosan-stimulated HoxB8 cells.

Volcano plots (middle) depict the expression change between the labeled comparisons (left). Members of the core inflammation program are colored in red, while the genes with the highest combined significance and effect sizes are labeled. Histograms (right) show the percentage of expression-matched background genes (as described in Fig. 5e and Methods). The red arrows indicate the observed percentage for core inflammation program members and is annotated with the respective overrepresentation *P*-value (one-sided, Wallenius method).

a Comparison of wildtype HoxB8 and HoxB8 carrying a *Cebpb* knockout in resting state. **b** Comparison of wildtype HoxB8 and *Cebpb*$^{-/-}$ HoxB8 after zymosan challenge. Source data are provided as a Source Data file.

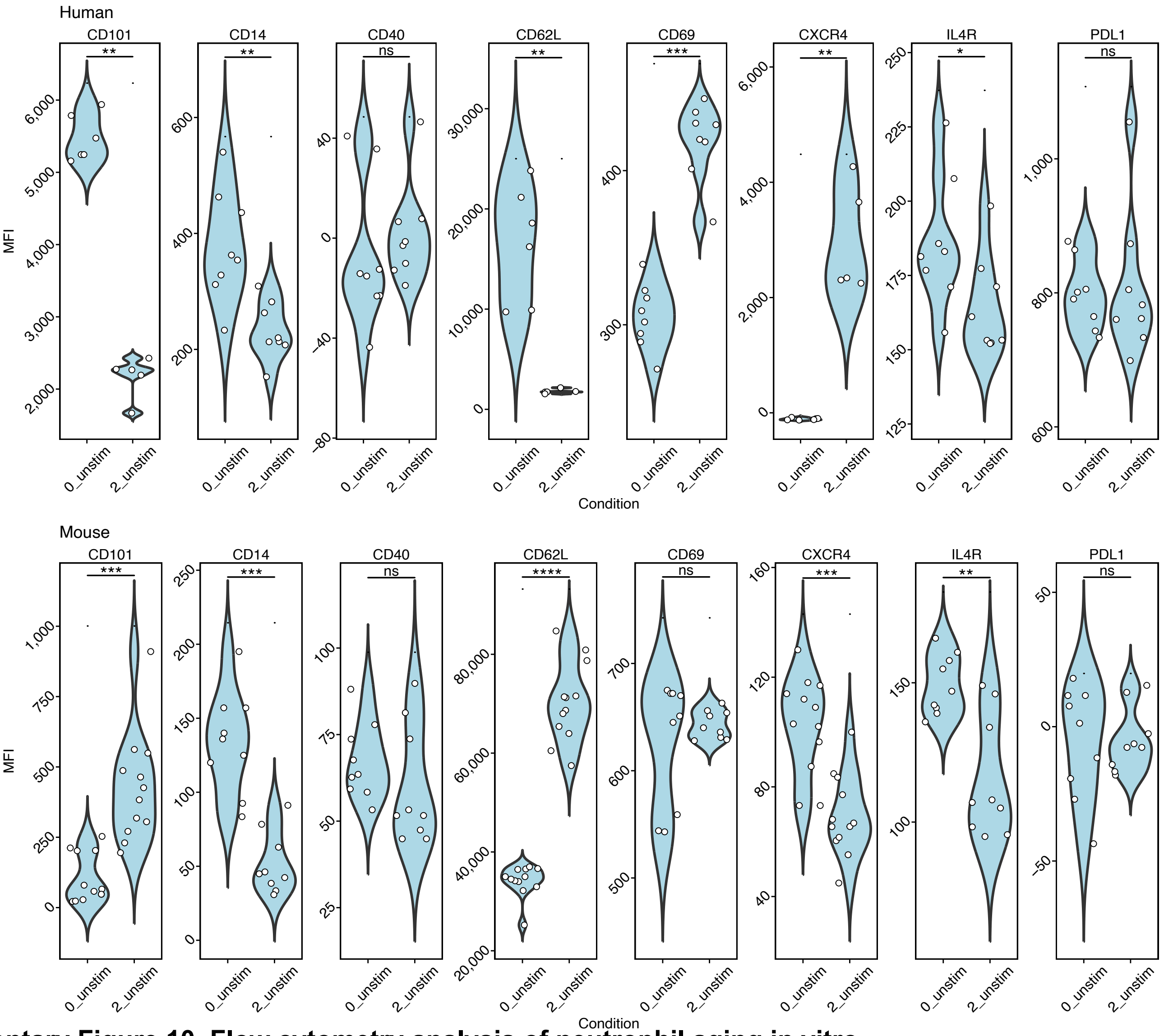

Supplementary Figure 10. Flow cytometry analysis of neutrophil aging in vitro.
MFI values of surface proteins depicted in Fig. 6c, with and without 2 days of cell culture. In addition, MFI values of CXCR4, CD62L, and CD101 derived from a different experimental series are shown. Source data are provided as a Source Data file.

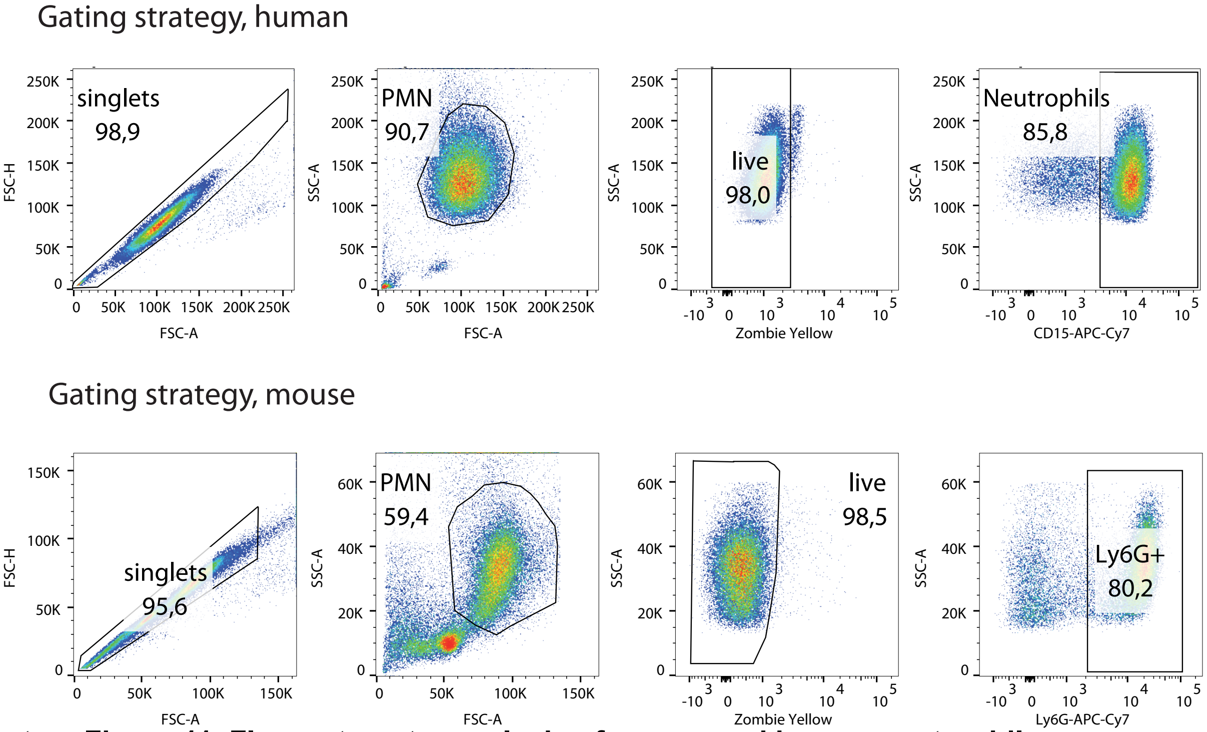

Supplementary Figure 11. Flow cytometry analysis of mouse and human neutrophils.
Gating strategy for human (top) and mouse (bottom) neutrophils.

REFERENCES

1. Ashburner, M. *et al.* Gene ontology: tool for the unification of biology. The Gene Ontology Consortium. *Nat Genet* **25**, 25-29 (2000).

2. Gene Ontology, C. The Gene Ontology resource: enriching a GOld mine. *Nucleic Acids Res* **49**, D325-D334 (2021).

3. Garcia-Alonso, L., Holland, C.H., Ibrahim, M.M., Turei, D. & Saez-Rodriguez, J. Benchmark and integration of resources for the estimation of human transcription factor activities. *Genome Res* **29**, 1363-1375 (2019).

4. Badia-i-Mompel, P. *et al.* decoupleR: ensemble of computational methods to infer biological activities from omics data. *Bioinformatics Advances* **2** (2022).

5. Patro, R., Duggal, G., Love, M.I., Irizarry, R.A. & Kingsford, C. Salmon provides fast and bias-aware quantification of transcript expression. *Nat Methods* **14**, 417-419 (2017).

6. Davis, S. & Meltzer, P.S. GEOquery: a bridge between the Gene Expression Omnibus (GEO) and BioConductor. *Bioinformatics* **23**, 1846-1847 (2007).
