# Supplementary material for: Human and mouse neutrophils share core transcriptional programs in both homeostatic and inflamed contexts
Source: Nat Commun. 2023 Dec 8;14:8133. doi: 10.1038/s41467-023-43573-9 (PMC10709367; doi:10.1038/s41467-023-43573-9)
Supplement: Supplementary file 3 — Description of Additional Supplementary Files [file 41467_2023_43573_MOESM3_ESM.pdf]

Description of Additional Supplementary Files

Supplementary Data 1. Quality control metrics for bulk RNA-seq data analysis.

Salmon[5] and FastQC quality control metrics after processing and mapping of raw sequencing reads. Provided are two sheets for resting and inflammatory analysis, and two sheets for the lineage analysis, separated by species, respectively.

Supplementary Data 2. List of studies included in the analysis.

One sheet per species. Metadata was retrieved using GEOquery[6] and curated manually to obtain consistency.

Supplementary Data 3. Results from Fisher's combined test.

Mean $\log_2$ fold change and results from Fisher's combined test generated in the analysis of inflammation, as outlined in Methods.
