# Supplementary material for: Human and mouse neutrophils share core transcriptional programs in both homeostatic and inflamed contexts
Source: Nat Commun. 2023 Dec 8;14:8133. doi: 10.1038/s41467-023-43573-9 (PMC10709367; doi:10.1038/s41467-023-43573-9)
Supplement: Supplementary file 7 — Reporting Summary [file 41467_2023_43573_MOESM7_ESM.pdf]

nature portfolio

Corresponding author(s): Ricardo Grieshaber-Bouyer

Last updated by author(s): Oct 5, 2023

Reporting Summary

Nature Portfolio wishes to improve the reproducibility of the work that we publish. This form provides structure for consistency and transparency in reporting. For further information on Nature Portfolio policies, see our Editorial Policies and the Editorial Policy Checklist.

Statistics

For all statistical analyses, confirm that the following items are present in the figure legend, table legend, main text, or Methods section.

n/a	Confirmed	
☐	☒	The exact sample size (*n*) for each experimental group/condition, given as a discrete number and unit of measurement
☐	☒	A statement on whether measurements were taken from distinct samples or whether the same sample was measured repeatedly
☐	☒	The statistical test(s) used AND whether they are one- or two-sided<br>*Only common tests should be described solely by name; describe more complex techniques in the Methods section.*
☐	☒	A description of all covariates tested
☐	☒	A description of any assumptions or corrections, such as tests of normality and adjustment for multiple comparisons
☐	☒	A full description of the statistical parameters including central tendency (e.g. means) or other basic estimates (e.g. regression coefficient) AND variation (e.g. standard deviation) or associated estimates of uncertainty (e.g. confidence intervals)
☐	☒	For null hypothesis testing, the test statistic (e.g. *F*, *t*, *r*) with confidence intervals, effect sizes, degrees of freedom and *P* value noted<br>*Give P values as exact values whenever suitable.*
☒	☐	For Bayesian analysis, information on the choice of priors and Markov chain Monte Carlo settings
☒	☐	For hierarchical and complex designs, identification of the appropriate level for tests and full reporting of outcomes
☐	☒	Estimates of effect sizes (e.g. Cohen's *d*, Pearson's *r*), indicating how they were calculated

Our web collection on statistics for biologists contains articles on many of the points above.

Software and code

Policy information about availability of computer code

Data collection	Publicly available data was retrieved using release 1.5 of the nf-core fetchngs pipeline. scRNAseq data were collected from the Sequence Read Archive (SRA) and European Nucleotide Archive (ENA). ATAC-sequencing data was retrieved from Gene Expression Omnibus. Flow cytometry data was collected as described below.
Data analysis	We provide analysis code for this study on GitHub: https://github.com/rgb-lab/inflamed_neutrophil_transcriptome<br>Key packages to reproduce our analysis are listed below.<br>Package name Version DOI or link<br>scanpy 1.9.2 https://scanpy.readthedocs.io/en/stable/index.html<br>magick 2.7.3 https://cran.r-project.org/web/packages/magick/index.html<br>DESeq2 1.36.0 https://doi.org/10.1186/s13059-014-0550-8<br>ggvenn 0.1.9 https://cran.r-project.org/web/packages/ggvenn/index.html<br>circlize 0.4.15 https://doi.org/10.1093/bioinformatics/btu393<br>ggsci 2.9 https://cran.r-project.org/web/packages/ggsci/index.html<br>RColorBrewer 1.1-3 https://cran.r-project.org/web/packages/RColorBrewer/index.html<br>viridis 0.6.2 https://cran.r-project.org/web/packages/viridis/index.html<br>viridisLite 0.4.0 https://cran.r-project.org/web/packages/viridisLite/index.html<br>MASS 7.3-57 https://cran.r-project.org/web/packages/MASS/index.html<br>rstatix 0.7.0 https://cran.r-project.org/web/packages/rstatix/index.html<br>ggbreak 0.0.9 https://cran.r-project.org/web/packages/ggbreak/index.html<br>reshape2 1.1.4 https://cran.r-project.org/web/packages/reshape2/index.html

kSamples 1.2-9 https://cran.r-project.org/web/packages/kSamples/index.html
SuppDists 1.1-9.7 https://cran.r-project.org/web/packages/SuppDists/index.html
gridExtra 2.3 https://cran.r-project.org/web/packages/gridExtra/index.html
ggrastr 1.0.1 https://cran.r-project.org/web/packages/ggrastr/index.html
ggtext 0.1.1 https://cran.r-project.org/web/packages/ggtext/index.html
EnhancedVolcano 1.14.0 https://doi.org/doi:10.18129/B9.bioc.EnhancedVolcano
ComplexHeatmap 2.12.0 https://doi.org/10.1093/bioinformatics/btw313
ggrepel 0.9.1 https://cran.r-project.org/web/packages/ggrepel/index.html
ggpubr 0.4.0 https://cran.r-project.org/web/packages/ggpubr/index.html
edgeR 3.38.1 https://doi.org/10.1093/bioinformatics/btp616
https://doi.org/10.1093/nar/gks042
https://doi.org/10.12688/f1000research.8987.2
limma 3.52.1 https://doi.org/10.1093/nar/gkv007
SummarizedExperiment 1.26.1 https://doi.org/doi:10.18129/B9.bioc.SummarizedExperiment
Biobase 2.56.0 https://doi.org/10.1038/nmeth.3252
GenomicRanges 1.48.0 https://doi.org/doi:10.18129/B9.bioc.GenomicRanges
GenomeInfoDb 1.32.2 https://doi.org/doi:10.18129/B9.bioc.GenomeInfoDb
IRanges 2.30.0 https://doi.org/doi:10.18129/B9.bioc.IRanges
S4Vectors 0.34.0 https://doi.org/doi:10.18129/B9.bioc.S4Vectors
BiocGenerics 0.42.0 https://doi.org/doi:10.18129/B9.bioc.BiocGenerics
MatrixGenerics 1.8.0 https://doi.org/doi:10.18129/B9.bioc.MatrixGenerics
matrixStats 0.62.0 https://cran.r-project.org/web/packages/matrixStats/index.html
biomaRt 2.52.0 https://doi.org/doi:10.18129/B9.bioc.biomaRt
BiocManager 1.30.18 https://doi.org/10.1038/nmeth.3252
zeallot 0.1.0 https://cran.r-project.org/web/packages/zeallot/index.html
magrittr 2.0.3 https://cran.r-project.org/web/packages/magrittr/index.html
forcats 0.5.1 https://cran.r-project.org/web/packages/forcats/index.html
stringr 1.4.0 https://cran.r-project.org/web/packages/stringr/index.html
dplyr 1.0.9 https://cran.r-project.org/web/packages/dplyr/index.html
purrr 0.3.4 https://cran.r-project.org/web/packages/purrr/index.html
readr 2.1.2 https://cran.r-project.org/web/packages/readr/index.html
tidyr 1.2.0 https://cran.r-project.org/web/packages/tidyr/index.html
tibble 3.1.7 https://cran.r-project.org/web/packages/tibble/index.html
ggplot2 3.3.6 https://cran.r-project.org/web/packages/ggplot2/index.html
tidyverse 1.3.1 https://doi.org/10.21105/joss.01686
org.Hs.eg.db 3.15.0 https://doi.org/doi:10.18129/B9.bioc.org.Hs.eg.db
TxDb.Hsapiens.UCSC.hg38.knownGene 3.15.0 https://doi.org/doi:10.18129/B9.bioc.TxDb.Hsapiens.UCSC.hg38.knownGene
GenomicFeatures 1.48.3 https://doi.org/10.1371/journal.pcbi.1003118
AnnotationDbi 1.58.0 https://doi.org/doi:10.18129/B9.bioc.AnnotationDbi
goseq 1.48.0 https://doi.org/10.1186/gb-2010-11-2-r14
geneLenDataBase 1.32.0 https://doi.org/doi:10.18129/B9.bioc.geneLenDataBase
BiasedUrn 1.07 https://cran.r-project.org/web/packages/BiasedUrn/index.html
vsn 3.64.0
survcomp 1.46.0 https://doi.org/10.1093/bioinformatics/btr511
prodlim 2019.11.13 https://cran.r-project.org/web/packages/prodlim/index.html
Matrix 1.4-1 https://cran.r-project.org/web/packages/Matrix/index.html
qvalue 2.15.0 https://doi.org/10.1073/pnas.1530509100
apeglm 1.18.0 https://doi.org/doi:10.18129/B9.bioc.apeglm
qusage 2.30.0 https://doi.org/doi:10.18129/B9.bioc.qusage
fgsea 1.22.0 https://doi.org/10.1101/060012
msigdbr 7.5.1 https://doi.org/10.1016%2Fj.cels.2015.12.004
sva 3.44.0 https://doi.org/doi:10.18129/B9.bioc.sva
BiocParallel 1.30.2 https://doi.org/doi:10.18129/B9.bioc.BiocParallel
genefilter 1.78.0 https://doi.org/doi:10.18129/B9.bioc.genefilter
mgcv 1.8-40 https://cran.r-project.org/web/packages/mgcv/index.html
nlme 3.1-157 https://cran.r-project.org/web/packages/nlme/index.html
GEOquery 2.64.2 https://doi.org/10.1093/bioinformatics/btm254
rhdf5 2.40.0 https://doi.org/doi:10.18129/B9.bioc.rhdf5
jsonlite 1.8.0 https://cran.r-project.org/web/packages/jsonlite/index.html
httr 1.4.3 https://cran.r-project.org/web/packages/httr/index.html
readxl 1.4.0 https://cran.r-project.org/web/packages/readxl/index.html
gridtext 0.1.4 https://cran.r-project.org/web/packages/gridtext/index.html
writexl 1.4.0 https://cran.r-project.org/web/packages/writexl/index.html
cowplot 1.1.1 https://cran.r-project.org/web/packages/cowplot/index.html
HOMER 4.11 https://doi.org/10.1016/j.molcel.2010.05.004
marge 0.0.4.9999 https://robertamezquita.github.io/marge/articles/marge-workflow.html#
RVAideMemoire 0.9-81-2 https://cran.r-project.org/web/packages/RVAideMemoire/index.html

For manuscripts utilizing custom algorithms or software that are central to the research but not yet described in published literature, software must be made available to editors and reviewers. We strongly encourage code deposition in a community repository (e.g. GitHub). See the Nature Portfolio guidelines for submitting code & software for further information.

Data

Policy information about availability of data

All manuscripts must include a data availability statement. This statement should provide the following information, where applicable:

- Accession codes, unique identifiers, or web links for publicly available datasets
- A description of any restrictions on data availability
- For clinical datasets or third party data, please ensure that the statement adheres to our policy

All sequencing data was used from publicly available platforms (Gene Expression Omnibus and European Nucleotide Archive), and all accession numbers are listed in Supplementary Data 2. Flow cytometry data have been deposited at flowrepository.org under the accession FR-FCM-Z6U3 and FR-FCM-Z6U4. Source data are provided with this paper.

Human research participants

Policy information about studies involving human research participants and Sex and Gender in Research.

Reporting on sex and gender	We collected samples from people of different sexes and genders. Sex- and gender-based analyses were not conducted as this was beyond the scope of the experiments in this particular study given the signal that we validated.
Population characteristics	Healthy volunteers participating in this study were 20–28 years old. Additional characteristics were not collected as this analysis was beyond the scope of the study given the biological signal that we validated in that section of the paper.
Recruitment	We recruited adult individuals without apparent current infection and without immunosuppressive medication. Individuals were selected randomly from a pool of co-workers. Potential biases include the confined age-range that could potentially limit the portability of the results to the biology of older individuals. Blood draws were restricted to the time window 8–10 AM to limit circadian variability.
Ethics oversight	IRB-approval Heidelberg-S-272/2021 obtained from the ethics committee of the University of Heidelberg, Heidelberg, Germany

Note that full information on the approval of the study protocol must also be provided in the manuscript.

Field-specific reporting

Please select the one below that is the best fit for your research. If you are not sure, read the appropriate sections before making your selection.

☒ Life sciences ☐ Behavioural & social sciences ☐ Ecological, evolutionary & environmental sciences

For a reference copy of the document with all sections, see nature.com/documents/nr-reporting-summary-flat.pdf

Life sciences study design

All studies must disclose on these points even when the disclosure is negative.

Sample size	For the meta-analysis, the datasets of interest were identified through a literature search on PubMed and the NCBI Gene Expression Omnibus. The number of included samples was limited by public availability of suitable RNA-sequencing samples.<br>For our confirmatory experiments, appropriate statistics including mean-size comparisons for our readouts of interests guided our sample size selection.
Data exclusions	6 RNA-Seq samples identified in our literature search were removed due to QC concerns. No flow cytometry samples were excluded from this study. We performed strict quality control in flow cytometry as stated in the gating strategy.
Replication	Flow cytometry was performed on samples collected from 14 human participants and 27 mice across individual experiments, defined as biological replicates. Experiments for Figures 6 and 7 were replicated in three independent experiments. All attempts at replication were successful. We used statistics including mean comparison were appropriate.
Randomization	All samples were cultured in the 3 conditions of interest, hence no randomization was applicable.
Blinding	For our flow cytometry experiments, statistical tests and comparisons were decided before data collection, no blinding was performed. In this study, homogenous groups were analyzed without group-comparisons that would require blinding. Downstream analysis after cell gating was performed using publicly available software with standard settings, which provides an unbiased approach towards the analysis and does not require blinding.

Reporting for specific materials, systems and methods

We require information from authors about some types of materials, experimental systems and methods used in many studies. Here, indicate whether each material, system or method listed is relevant to your study. If you are not sure if a list item applies to your research, read the appropriate section before selecting a response.

Materials & experimental systems

n/a	Involved in the study
☐	☒ Antibodies
☒	☐ Eukaryotic cell lines
☒	☐ Palaeontology and archaeology
☐	☒ Animals and other organisms
☒	☐ Clinical data
☒	☐ Dual use research of concern

Methods

n/a	Involved in the study
☒	☐ ChIP-seq
☐	☒ Flow cytometry
☒	☐ MRI-based neuroimaging

Antibodies

Antibodies used

Human flow cytometry panel:
LIVE/DEAD Pacific Orange N/A BioLegend 423103 1:300
LIVE/DEAD BUV496 N/A BioLegend 423107 1:300
DAPI BUV496 N/A Sigma-Aldrich D9542 1:1000
CD15 APC-Cy7 W6D3 BioLegend 323047 1:100
CD69 BV421 FN50 BioLegend 310929 1:50
CD40 Alexa Fluor 700 5C3 BioLegend 334327 1:50
CD14 PE-Cy7 M5E2 BD Biosciences 557742 1:100
IL4R PE G077F6 BioLegend 355003 1:100
PD-L1 APC 29E2A3 BioLegend 329708 1:20
CD101 APC BB27 BioLegend 331007 1:100
CXCR4 BV421 12G5 BioLegend 306517 1:100
CD62L BV605 DREG-56 BioLegend 304833 1:100

Mouse flow cytometry panel:
Marker Channel Clone Vendor Catalog# dilution
LIVE/DEAD Pacific Orange N/A BioLegend 423103 1:300
LIVE/DEAD BUV496 N/A BioLegend 423107 1:300
DAPI BUV496 N/A Sigma-Aldrich D9542 1:1000
Ly6G APC-Cy7 1A8 BioLegend 127623 1:100
CD69 BV421 H1.2F3 BioLegend 104527 1:100
CD40 PE-Cy5 3/23 BioLegend 124617 1:100
CD14 PE-Cy7 Sa14-2 BioLegend 123315 1:100
IL4R PE I015F8 BioLegend 144803 1:100
PD-L1 APC 10F.9G2 BioLegend 124311 1:100
CD101 APC Moushi101 ThermoFisher Scientific 17-1011-82 1:100
CXCR4 BV421 L276F12 BioLegend 146511 1:100
CD62L BV605 MEL-14 BioLegend 104437 1:100

Validation

Antibodies only included established, well validated clones and were validated by the manufacturer (details on the product page for each antibody, see catalog numbers above). No additional antibody validation was performed.

BioLegend obtained antibodies: Manufacturer's quality control includes specificity testing of 1-3 target cell types with either single- or multi-color analysis (including positive and negative cell types) with the evaluation of brightness for negative and positive cell populations in comparison to reference lots. Each product is validated by QC testing with a series of dilutions.

BD Biosciences obtained antibody: BD Biosciences established reference standards for all antibodies and when new lots are produced, they are compared to this reference (gold standard). The new lots must match the performance with regards to the quality control (QC) specifications that were set for that given antibody (clone and format). They do not release our QC specifications. (personal correspondence)

All specified antibodies were verified to show reactivity towards their respective antigen in human or mouse. For anti-human CXCR4 (BioLegend 306517) and anti-human CD40 (BioLegend 334327), species reactivity of Cynomolgus and Rhesus were verified). All specified antibodies were validated for surface staining in flow cytometry applications as used in this study.

Animals and other research organisms

Policy information about studies involving animals; ARRIVE guidelines recommended for reporting animal research, and Sex and Gender in Research

Laboratory animals	Mus musculus, C57BL/6J, 8-12 weeks
Wild animals	No wild animals were used in this study
Reporting on sex	Both female and male mice were used. Sex-based analyses were not conducted as this was beyond the scope of the experiments in this particular study given the signal that we validated.
Field-collected samples	No field collected samples were used in this study.
Ethics oversight	Approval of the Animal Care Facility Heidelberg and the Animal Welfare Officers (approval #T66/21) at the Interfaculty Biomedical Research Facility (IBF), University of Heidelberg, Germany

Note that full information on the approval of the study protocol must also be provided in the manuscript.

Flow Cytometry

Plots

Confirm that:

- [x] The axis labels state the marker and fluorochrome used (e.g. CD4-FITC).
- [x] The axis scales are clearly visible. Include numbers along axes only for bottom left plot of group (a 'group' is an analysis of identical markers).
- [x] All plots are contour plots with outliers or pseudocolor plots.
- [x] A numerical value for number of cells or percentage (with statistics) is provided.

Methodology

Sample preparation	Human: Peripheral blood of healthy donors was collected (IRB-approval: Heidelberg-S-272/2021). Neutrophils were isolated using density gradient centrifugation with Polymorphprep, 30 ml whole blood was layered onto 20 ml Polymorphprep and centrifuged at 535 g for 35 minutes. The PBMC-containing layer was discarded by suction and neutrophils were recovered and subjected to hypotonic lysis using 0.2% NaCl. The cells were subsequently washed with cell culture medium (RPIM-1640 + 10% FBS + 1% GlutaMAX) and seeded at 5 million cells per 6 wells in a total volume of 5 ml at a humified atmosphere at 37° C with 5% CO2. The cells were cultered over 48h either in the absence of cytokines (vehicle control), with GM-CSF + IFN-gamma or GM-CSF + LPS. GM-CSF was used at a final concentration of 100 U/ml, IFN-gamma at 10 ng/ml and LPS at 100 ng/ml. After 48h, 1 million cells were collected and stained using the Zombie Yellow Fixable Viability Kit for live/dead discrimination, followed by an antibody panel in 50µL of FACS buffer (2% FBS, 5mM EDTA and 0.1% sodium azide in PBS for 25 minutes.<br>Mouse: Male and female C57BL/6J mice were sacrificed by cervical dislocation and bone marrow was extracted by flushing with RPMI. Neutrophils were enriched by density centrifugation using Histopaque 1077 and Histopaque 1119. Cells were recovered from the interphase of both Histopaque layers and centrifuged. Cells were washed with RPMI containing 10% FBS and 1% Glutamax and seeded at 106 cells/ml in 48 well plates in a total volume of 500 µl. Murine GM-CSF, murine IFN-γ and LPS were added to the medium for 24 hours and 48 hours in combination as indicated in the respective figures. Cells cultured in the absence of cytokines were used as controls. After the indicated times, cells were collected and stained with the antibody panel in 50 µl of FACS buffer containing 2% FBS, 5 mM EDTA and 0.1% sodium azide.<br>To assess neutrophils from different organs, mice were sacrificed by cardiac puncture under generalized anesthesia. Subsequently, the femora and tibiae were flushed with PBS to obtain bone marrow. Any remaining fat was removed from spleens and splenic tissue was mechanically disintegrated using the back of a syringe. Cells were pelleted at 400 g and erythrocytes were lysed using ACK Lysing Buffer for 5 minutes at 4°C. Cells were seeded at 10^6 cells/ml in 48 well plates in a total volume of 500 µl. Cytokines were added as described above for a total of 8 hours.
Instrument	Flow cytometry was performed on an BD LSRII flow cytometer.
Software	Flow cytometry files were analyzed using BD FACSDiva version 8.0.1 and FlowJo version 10.8.0.
Cell population abundance	The population of interest (CD15-APC-Cy7 positive in human or Ly6G-APC-Cy7 in mice) exceeded fraction of 75% living cells in our analyses as defined in the gating strategy.
Gating strategy	Cells were gated for singlets in FSC-A/FSC-H, for PMN's by FSC-A/SSC-A, living cells by Zombie Yellow/ZombieUV/DAPI, and neutrophils by CD15-APC-Cy7 in human and by Ly6G-APC-Cy7 in mice.

- [x] Tick this box to confirm that a figure exemplifying the gating strategy is provided in the Supplementary Information.
